# Understanding social context in HIV and drug use via community-informed computational modeling: SILOS study protocol

**DOI:** 10.3389/fpubh.2025.1690612

**Published:** 2025-12-04

**Authors:** Joshua Z. Stadlan, Patrick F. Janulis, Tom Wolff, Kathryn Risher, Jonathan Ozik, Sara P. Rimer, Elizabeth A. McConnell, Darnell Motley, Gregory Phillips, Kate Banner, Joshua Melville, Caden Buckhalt, Emily Esposito, Michelle Birkett

**Affiliations:** 1 Department of Medical Social Sciences, Feinberg School of Medicine, Northwestern University, Chicago, IL, United States; 2 Northwestern Institute on Complex Systems, Northwestern University, Evanston, IL, United States; 3 Department of Public Health Sciences, College of Medicine, Penn State University, Hershey, PA, United States; 4 Argonne National Laboratory, Lemont, IL, United States; 5 Department of Public Health Sciences, The University of Chicago, Chicago, IL, United States; 6 Northwestern-Argonne Institute for Science and Engineering (NAISE), Northwestern University, Evanston, IL, United States; 7 Consortium for Advanced Science and Engineering (CASE) at the University of Chicago, Chicago, IL, United States; 8 Department of Psychology, Palo Alto University, Palo Alto, CA, United States; 9 Department of Medicine, The University of Chicago, Chicago, IL, United States

**Keywords:** HIV, sexual networks, social networks, participatory modeling, race/ethnicity, disparities, systems science, social determinants of health

## Abstract

**Introduction:**

Racial, ethnic, and sexual and gender minority populations are disproportionately impacted by HIV and other infectious diseases. A rigorous and holistic view of how individual and complex factors interact to produce health disparities among minority populations is urgently necessary.

**Methods:**

This project seeks to understand the social and contextual systems around the most marginalized HIV-impacted populations by investigating how differences in access to people and places confer HIV risk. Specifically, we have planned an innovative observational study across five US cities, administering in-depth remote network surveys to 2,700 racially diverse young men who have sex with men and transgender women. The data captured will be used to develop simulation models of city-specific synthetic populations to examine how differences in the people and places populations have access to pools risk within marginalized populations and increases their disparities in HIV.

**Results:**

The study was awarded in August 2024 but has paused since March 2025 due to the NIH’s termination of the study just prior to the start of participant recruitment. The future of the work is currently unclear as we pursue multiple avenues for reinstatement.

**Discussion:**

Drawing upon both our expertise in network modeling and our strong community partnerships, this project remains poised to transform scientific understanding of the structural drivers of health inequity.

## 1 Introduction

### 1.1 Background on HIV disparities

Racial, ethnic, and sexual and gender minority populations are disproportionately impacted by HIV and other infectious diseases, with Black men who have sex with men (BMSM) and Black transgender women (TW) bearing a disproportionate burden (1–3).

Black individuals in particular experience some of the highest rates of HIV, followed by Hispanic/Latinx populations, with these groups experiencing rates of 41.1 and 16.1 infections per 100,000 population (respectively); by percentage, Black and Hispanic/Latinx populations accounted for approximately 43 and 24% (respectively) of all new diagnoses in the United States (4).

Furthermore, sexual minorities–specifically men who have sex with men (MSM)–are disproportionately impacted by HIV, accounting for over 70% of all new diagnoses per the same CDC report (4). Finally, those at the intersection of these two groups appear to have their disparities magnified (4–6). The same recent CDC data shows that, of all new HIV infections in males in the US and dependent areas, new infections in males in the US and dependent areas attributable to male-to-male sexual contact, BMSM represented just over 38% of all new diagnoses among MSM, followed by Hispanic/Latinx MSM at just over 28%. Meanwhile, White MSM accounted for just over 27%, despite non-Hispanic/Latinx White Americans representing 58% of the population (4, 5, 7). These data reflect a consistent pattern of disparities observed by race, ethnicity, and sexual minority status (4–6).

TW are also disproportionately impacted by HIV, with a recent CDC report finding approximately 42% of surveyed transgender participants to be living with HIV (8). Again, major differences in HIV by race were found among transgender participants, with 62 and 35% of Black and Hispanic/Latinx TW (respectively) living with HIV compared to only 17% of White TW (8).

Substance use–an important driver of HIV through elevated sexual risk behaviors and biomedical prevention interference–is also substantially elevated within MSM and TW (8–10). For example, gay men report rates of illicit drug use 1.89–2.30 times higher than heterosexual men across all ages 18–49 (11). This disparity is even greater for methamphetamine use, which is reported 3.76 times higher in gay compared to heterosexual men (11). Finally, 45.3% of gay men report any lifetime sexualized drug use (i.e., chemsex) indicating a sizable minority of MSM use substances in combination with sexual activity (12). Recent studies suggest that for populations facing numerous intersecting stigmas, substance use serves as a coping mechanism and a way of facilitating both connection and sexual pleasure (10).

Researchers have yet to synthesize a rigorous and holistic view of how individual and complex factors interact to produce health disparities among minority populations. Across a broad range of health outcomes, the disparities experienced by racial, ethnic, and sexual minority populations are believed to be caused by differences across a multitude of factors–from housing (10, 13–15), employment (16, 17), academic outcomes (18–20), social relationships (13, 21), and health care (13, 14)–but little is understood about how these differences in social and structural factors manifest, or by what specific pathways they determine health. Within HIV specifically, the individual-level risk factors typically associated with the disease are not predictive of disparities, as several meta-analyses demonstrate that BMSM have fewer high-risk sexual behaviors, fewer sexual partners, less illicit drug use than White MSM (22–24) as well as greater engagement in HIV preventive behavior (22).

Therefore, understanding the drivers of health disparities requires a novel approach–one which seeks to understand the social and contextual systems around the most marginalized populations–as it is through an individual’s interactions with their social context that any advantage or disadvantage is conferred.

### 1.2 Structural accounts of HIV disparities

#### 1.2.1 Networks and HIV

Several features of the sexual networks of BMSM have been used to explain the persistence of racial disparities in HIV despite similar or lower rates of individual risk behavior (12, 22). First, people living with HIV are more likely to have older Black partners (12, 22, 25–27). Likewise, BMSM are especially likely to partner with other BMSM (26, 28–32). Therefore, the higher prevalence of HIV among partners of BMSM may itself help perpetuate the spread of HIV in this population (29, 33, 34). These patterns persist within Hispanic/Latinx MSM: White Hispanic/Latinx primarily partner with White MSM, Black Hispanic/Latinx with BMSM (29, 30). Racial mixing patterns are exceptionally important in understanding HIV disparities; however, it must be noted that network simulation has demonstrated that racial mixing patterns alone are unable to either create or sustain racial disparities in HIV–therefore other mechanisms must be examined (31).

Another potential driver of disease is having concurrent sexual partners, as even small increases in concurrency, or sex tie overlap, can significantly decrease network path length and allow for greater disease transmission (35, 36). However, within MSM there has been no evidence of racial differences in sexual partner concurrency (28, 29, 37). That said, racial differences in sexual network structure persist (28, 29, 37, 38). For example, network simulations conducted by our team have demonstrated that compared to White MSM, BMSM have a greater likelihood of being in a sexual network component with an individual living with HIV, and were also a shorter distance to these individuals when in such a component (29).

Several studies have shown that people living with HIV are likely to cluster together socially and not just sexually (29, 39–41), echoing numerous sociological studies demonstrating that similar individuals tend to form connections with one another (i.e., “birds of a feather flock together”). However, mechanisms driving this clustering–commonly termed “homophily”–are often extremely complicated (i.e., socialization, selection, or opportunity structures) (42–44). Accordingly, a better understanding of the multilevel mechanisms that connect certain populations and concentrate risk is necessary to yield effective insight to combat HIV disparities.

#### 1.2.2 Places and HIV

##### 1.2.2.1 Neighborhood

Increasingly, physical spaces such as neighborhoods have been examined to explain racial HIV disparities, both in conjunction with and independent of network features. For example, in our previous study of 450 MSM, we found that two characteristics of the built environment (walkability and vacant housing) were significant predictors of HIV status, while individual-level risk behaviors such as condomless anal sex and drug use were not (45). Another study of HIV racial disparities within MSM found that the only individual-level factor that reduced the adjusted hazard ratio (aHR) for HIV between Black and White MSM was health insurance status, while two neighborhood-level factors significantly reduced the aHR (living in a predominantly Black neighborhood, living in a neighborhood with a low median income) (26, 45). Further, HIV prevalence differs substantially across neighborhoods. Chicago data demonstrates that HIV prevalence and incidence were concentrated in a few community areas which also tended to have the highest scores on a measure of community disadvantage (46). Finally, through a mixed methods investigation of Chicago MSM, we found several neighborhood-level patterns likely to create opportunity structures that shape and segregate HIV risk environments by race, including racial segregation and resource inequality, White neighborhood spatial insularity, and Black and Hispanic/Latinx neighborhood bridging (47).

##### 1.2.2.2 Physical and online venues

Sexual- and gender minority-specific venues have been identified as important social places for MSM and TW to engage in risk behaviors outside of their residential neighborhood (48, 49), and are increasingly being examined as drivers of risk disparities. For example, one study found that BMSM were highly connected around a small number of venues, indicating that partner-seeking patterns would likely create densely connected sexual networks in this population (50). Similarly, seeking partners through mobile applications is very common (51) and may create parallel dynamics given the high levels of racial homophily observed in these spaces and racial segregation across specific applications (52).

These network-based and place-based analyses have advanced our understanding of HIV disparities beyond individual behavior-based analyses by incorporating social context. However, we still lack an understanding of the mechanism driving disparities, how individual behaviors of populations interact with social and contextual factors at scale, and how public health might most appropriately intervene. Therefore, innovative approaches are necessary to holistically investigate how the structural inequities experienced by marginalized populations likely drive disparities in HIV. By moving away from traditional frameworks of data collection, measurement, and analysis which focus only on individuals and their behaviors, this study is able to examine how the entire system fits together to produce the health of a particular population. Specifically, our focus on understanding the social context around individuals–or the people and the places that individuals are connected to–as well as our use of system science methods allow us a unique opportunity to examine how structural inequities may impact access to resources and exposure to risk across populations.

#### 1.2.3 A system science approach

Population level disparities, by definition, are only quantifiable at the population level. Health, on the other hand, is generally conceptualized on the individual level. While drivers of population disparities may operate through differences at the individual level, they are unlikely to be particularly visible or significant at that level. A systems science approach acknowledges the interconnection of phenomena across scales and explores how behavior at certain scales can induce phenomena on other scales beyond the sum of the constituent behaviors (emergence) (53). Within this study we investigate how disparities emerge from differences across individuals, their social networks, the places in which they spend time, and how these differences scale across entire populations. We model these differences by pairing the city-specific empirical data collected across each of these levels with derived synthetic populations built to map onto the demographics of each city. This approach allows us to build sophisticated models of how these populations interact over time. While our approach is sophisticated computationally, we are simultaneously focused on balancing our computational approach with the meaningful engagement of local stakeholders. Community engagement is an important method for injecting realism into modeling, as those with analytic expertise often lack real-world understanding. It also ensures that findings are presented and interpreted in accordance with local understanding, and that any suggested interventions based on findings are compelling and feasible for local stakeholders. Through the assembly of five city-specific Community Advisory Boards, as well as the leadership of five Community Engagement Advisors, this study builds on our team’s established history of collaboration with local community organizations–local public health departments, community-based organizations, and community advisory boards whose members are recruited with the intention of promoting diverse perspectives.

### 1.3 Objectives

In this protocol paper, we present the proposed method of the study SILOS: Understanding Structural Inequities across Layers Of Social-Context as Drivers of HIV and Substance Use.

We propose innovative observational research across five US cities to better understand the social contexts of racial, ethnic, and sexual and gender minority populations, as well as how inequities in social contexts drive HIV and substance abuse. Specifically, through in-depth remote network surveys of 2,700 racially diverse young men who have sex with men and transgender women (YMSM-TW), we will examine how an individual’s social position determines the people and the places they have access to, as well as how supportive or risky these social and contextual environments are and how these connections might pool risk and provide fewer resources to those with marginalized and multiple marginalized identities. Uniting sophisticated network modeling with a robust plan for community engagement, this project is well-suited to transform scientific understanding of the structural drivers of health inequity. Our aims are threefold:

**Aim 1: Document structural inequities by identity.** Empirical observations of the social relational and contextual connections of racially diverse YMSM-TW will be captured and inequities across identity examined.

**Aim 2: Examine structural inequity as a fundamental driver of disparities in HIV and Substance Use.** Simulation and network modeling will be used to test if inequities across social contextual structures account for disparities in HIV, over and above identity alone.

**Aim 3: Conduct city-specific analyses.** We will explore differences in the experiences of YMSM-TW across cities.

To carry out these aims and evaluate these hypotheses, we devised a method that bridges individual, social relational, social contextual, and population health scales, and incorporates community perspectives into computational modeling, which we present in this protocol paper. This paper proceeds as follows. We divide the next section, Methods and Analysis, into four parts: first, we review the overall study design; second, we discuss our community engagement approach; third, we describe network interview-based data collection; fourth, we outline our quantitative analysis and computational modeling plan, further grouped by study aim. Then, we proceed to discussing the protocol’s strengths and limitations–on both the programmatic and scientific level. We conclude with the study’s dissemination plan and ethical considerations.

## 2 Methods and analysis

### 2.1 Overview of study design

SILOS will conduct in-depth remote network interviews of 2,700 racially diverse YMSM-TW across five cities (i.e., New York City, Chicago, Houston, Atlanta, Miami), capturing each participant’s personal networks of ties to other people and to places related to social position and HIV risk. Human networks collected will focus on social support ties and sexual partnerships and will be supplemented by response items related to social status and substance use. Place networks collected will focus on the physical venues in which participants socialize, sexual networking apps participants use, and health care providers participants access. Community Engagement Advisors and local Community Advisory Boards in each city will steer recruitment and provide neighborhood context for the processing and analysis of place networks.

While Aim 1 analyses will draw from the empirical data captured, in order to make population-level inferences Aims 2 and 3 will match captured empirical data on the people and places individuals are connected to with city-specific, demographically representative synthetic populations. This will be used to calibrate an adaptive network model of sexual partnership and HIV transmission, as well as simulate interactions with physical venues and online apps. The resulting model is a spatially embedded agent-based model with network-based epidemic dynamics capable of large-scale simulation experiments on the causal relationship between social factors and HIV epidemic trends.

**Figure 1:**
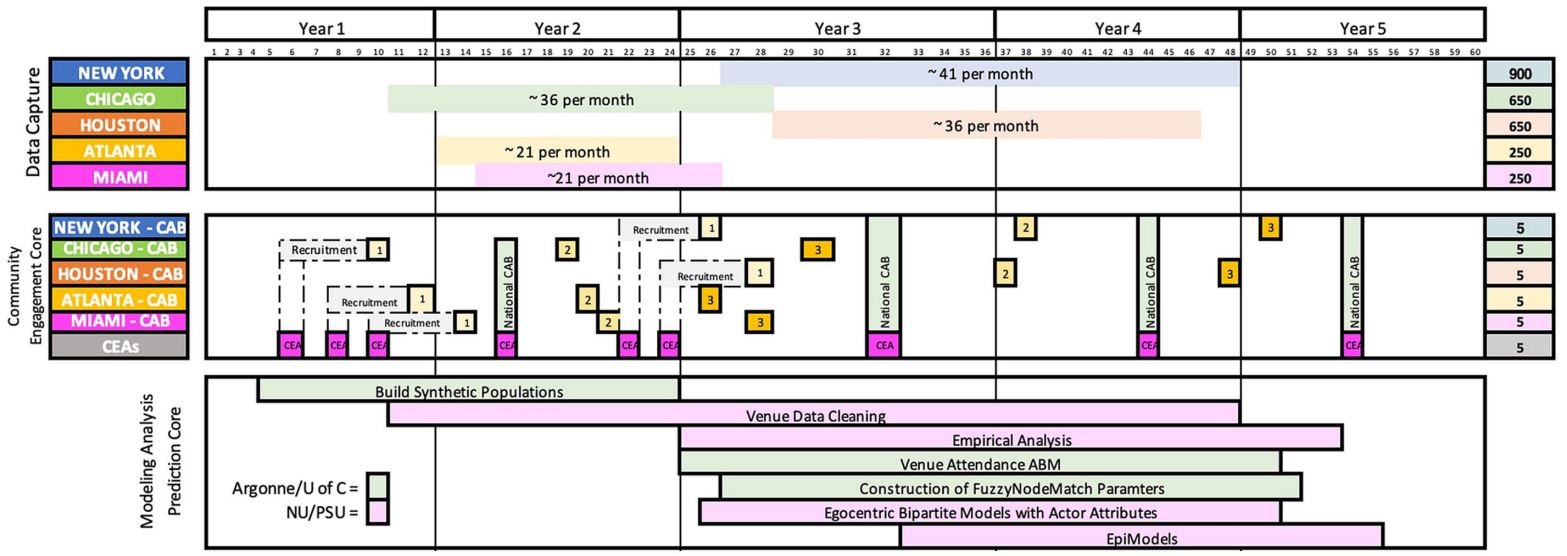
Overall study structure and schedule.

We provide more detail about the community engagement, network interviews, and quantitative analysis components of the protocol below. Figure 1 sketches the overall proposed schedule of these three streams of the study.

### 2.2 Community engagement core

We have included five Community Engagement Advisors (CEAs) as key personnel on the study, themselves formal and informal social leaders in study-relevant groups such as the national House Ballroom scene. These individuals have been key in guiding all aspects of our proposal and will be essential for shaping our team’s national strategy for community engagement.

**Figure 2:**
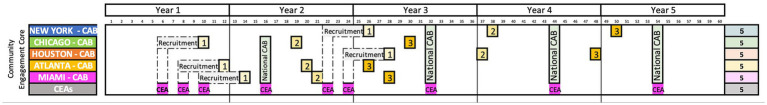
Activities of the community engagement core.

Further, we will develop five city-specific community advisory boards (CABs) of six racially diverse YMSM-TW (i.e., 30 total individuals). Local CABs will be essential in guiding the study recruitment strategy within each city and will provide a local perspective on study data–in particular the cleaning of places data–to help guide model development throughout the duration of the project. CAB members will have opportunities to serve as study coauthors and/or assist in other dissemination activities. Within each city, we anticipate convening three two-hour long virtual local CAB meetings. Figure 2 presents our proposed CAB schedule. The first meeting will discuss study recruitment strategy, the second to discuss data cleaning of places data, the third to discuss our initial analyses and the findings of city specific data. In addition to these city-specific meetings, beginning in our second year, we will also convene an annual Virtual National CAB Meeting (inclusive of all CAB members as well as CEAs).

Recruited CAB members will identify as either Black or Hispanic/Latinx (or both), will be between the ages of 18 and 29, and will either identify as MSM or TW. We intentionally will not include non-Hispanic/Latinx White MSM within our CABs to focus on the experiences of racial minority individuals who are most impacted by HIV. Based on previous studies’ participation trends, opening the CAB to non-Hispanic/Latinx White MSM risks saturation of participation by the representatives of lower-impacted demographics, introducing a greater risk of bias than restricting CAB membership. Non-Hispanic/Latinx White MSM are already over-represented in the relevant research literature, so reliance on previous published findings about MSM behavior mitigates concern of potential knowledge gaps induced by restricting the CAB to Black and Hispanic/Latinx identities.

Also, we will recruit at least one and up to two TW CAB members per city, as they are at the intersection of multiple marginalized identities and experience significant disparities in HIV.

We will implement an anonymous feedback channel for CAB members to the projects’ co-investigators specializing in community engagement to monitor for any concerns. We will record attendance and note retention trends, but due to the small size of the CABS (six persons each) and limited number of sessions (three each), we will rely on qualitative observation to assess participant engagement, satisfaction, and incorporation of feedback.

### 2.3 Network interviews

#### 2.3.1 Target sample

A total of 2,700 racially diverse young (18- to 29-year-olds) MSM and TW will be recruited within this study, with an overall racial/ethnic distribution of 28% Black; 30% White; 32% Hispanic/Latinx, 10% Other. We set separate target samples for each of the five cities, proportional to the racial/ethnic demographics of that age bracket in the city, according to U. S. Census tables. Recruitment target sizes were also set to approximate 5% of the estimated YMSM-TW population within each city (54), replicating the size of our previous data collection [PLoT ME: Plotting Layers of Transmission in Micro-Epidemics; a study limited to Chicago (55)], with some adjustment: we scaled them to account for the immense differences in population density across the cities. For example, we increased the targeted sample size within Houston so that at least one empirical observation would be captured per square mile of land area.

In determining the sample size targets, we also conducted a statistical power analysis on the detection of differences in demographic comparisons. We anticipate sufficient power as even within the smallest city-specific sample (i.e., 250 individuals within Atlanta), we expect 123 Black YMSM-TW and 99 White YMSM-TW; as such we will have 86.8% power to detect an incidence rate ratio of 1.6 or greater in network degree differences across these two groups, using Poisson regression.

Recruitment will begin at the end of Year 1 and continue through Year 4.

#### 2.3.2 Recruitment

Recruitment efforts will include place and event-based advertising (e.g., LGBTQ+ affinity spaces, SGM-focused health care providers, and House Ball events), advertising campaigns run on public transportation (e.g., buses, trains) in targeted areas across the five target cities, targeted online advertising on social media platforms (e.g., Facebook, Instagram, TikTok). Our *Community Engagement Core*–in particular our CEAs and our local city-specific CABs–will be essential in helping us to identify appropriate spaces, organizations, and community-based outreach events where we will disseminate recruitment materials.

All materials will include QR codes that will link to our screener while online advertisements will directly link. Given our study’s focus on understanding the racial differences in the use of online hook-up apps, we will intentionally not purchase ad-buys on hook-up apps–particularly those which are highly racially stratified (Grindr) (54, 55)–as to not bias our observations. Each participant who completes the Network Canvas survey will be compensated with a $50 VISA Gift Card.

#### 2.3.3 Survey instrument

**Figure 3:**
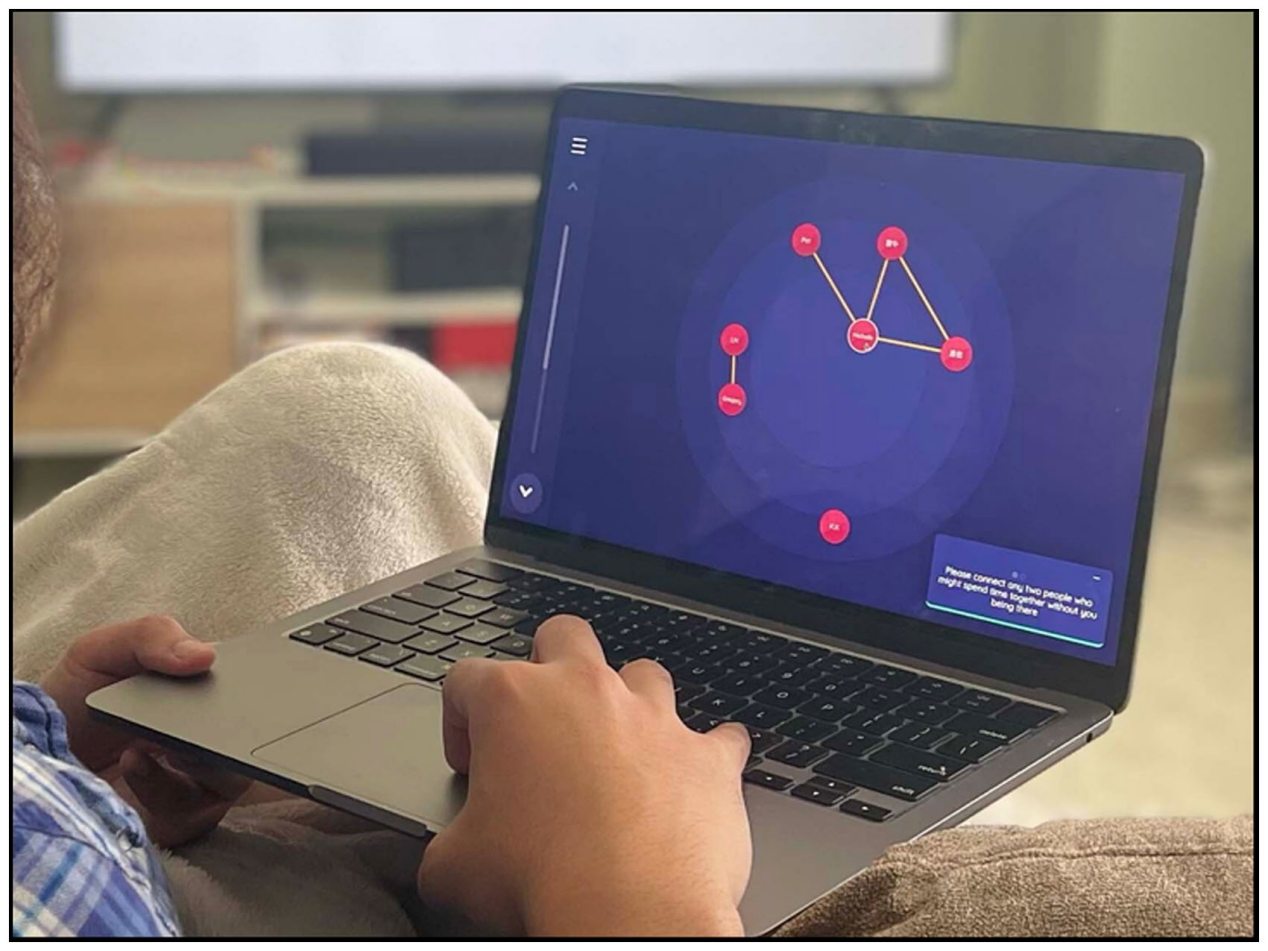
Participant completing a network canvas interview.

Participants who consent to participate in the study will complete a one-time online survey within Network Canvas on either a personal computer or tablet, as illustrated in Figure 3. Network Canvas (27) is an NIH-funded software developed by members of our team, designed to capture social network data through visual and intuitive survey interfaces. To date, Network Canvas has been deployed in at least 52 NIH-funded studies and has demonstrated higher-quality data collection compared to other survey tools (56–58). Network Canvas is a powerful tool for implementing “hypernetwork” survey methods that link individuals not just to people, but also to groups and places, making it especially useful for capturing location data through surveys.

The self-administered network survey will capture data at three levels–individuals, individual connections to people, and individual connections to places. We have included a draft of the Network Canvas survey instrument–a Network Canvas file and summary PDF–in the Supplementary material.

##### 2.3.3.1 Individual

At the individual-level, data will be captured on participant’s self-reported demographics (e.g., race/ethnicity, gender, sexual orientation, socioeconomic status, residence and residence history) and health and health behaviors [e.g., HIV status, HIV testing, pre-exposure prophylaxis (PrEP)], substance use (e.g., use of marijuana, cocaine, heroin, opiates, methamphetamines, and poppers), individual perceptions of social support, and internalized homophobia. As how others perceive an individual’s identity also shapes their social position, we will also query individuals about their perceptions on how others view them across race/ethnicity, gender, sexual orientation, and socioeconomic status (SES).

##### 2.3.3.2 Connections to people

We will query participants about two of their people networks: individuals who provide them social support (Social Network Members) and individuals with whom they have had sex (Sexual Network Members). The network interview consists of three steps: name generation, name interpretation, and edge generation (59). In the name generation step, participants add members to each network. We will use the name generators–the questions that specify which people should be considered network members–that we have successfully used in prior studies with YMSM-TW (55). Study participants will be able to enter either a first name or nickname for their network members, but this will only serve to assist the participant in answering questions about their network and it will not be used for our analysis. Next, in the name interpretation step, important attributes of these social and sexual connections will be queried. Participant perceptions of the demographics of their network members (race/ethnicity, sex, gender, sexual orientation, SES, neighborhood of residence) will be queried as well as how the network member was met, the strength of relationship, and perceived substance use. For sexual partners, important aspects of their sexual relationship relevant to accurately modeling HIV transmission will be assessed (e.g., partnership type, estimated dates of first and most recent sex, perceived HIV status, condom use, use of substances during sex, where partner was met). Finally, in the edge generation step, participants will be shown a sociogram–an interactive map of the network members–where they can indicate the social and sexual ties among their network members.

##### 2.3.3.3 Connections to places

We will query participants about three types of places–physical venues they attend (Physical Venues), online apps they use (Online Venues), and Health Care Providers they access (Health Care Providers). Similar to the capture of social network data, we will utilize network generators for building lists of physical venues, online venues, and health care providers. We will use place name generators successfully utilized in prior studies (55). When relevant, approximate geographic location of the specific places will be captured (Neighborhood). Participants will also be asked about their frequency of attendance and participant perceptions of clientele, and perceptions of venue patron drug use.

### 2.4 Analysis

We will use descriptive statistics, network models, agent-based models, and calibrated stochastic epidemic models to investigate our hypotheses. We discuss these methods for each aim of the project.

#### 2.4.1 Aim 1: documenting structural inequities by identity

Empirical observations of the social relational and contextual connections of racially diverse YMSM-TW will be captured and inequities across identity examined. We hypothesize that: *(**H1**) those who are and who are viewed as multiple marginalized individuals will have the fewest connections, whether to people like social supports or sexual partners, or to physical and online places where partners are met or to health care providers; (**H2**) multiple marginalized individuals will have to travel farther to access these people or places*; *(**H3**) sexual network and co-location mixing patterns will* *vary so marginalized and multiple marginalized individuals are clustered together and segregated from majority populations*.

Toward examining H1 and H2, we will first produce descriptive statistics of the Network Canvas data. We will characterize, and compare across racial/ethnic and gender identities, several attributes of respondents (egos): the degree of their social and sexual networks, the number of places (Physical Venues, Online Venues, Health Care Providers) they nominate, and the distance they travel to those places (given the home location of the ego and their partners). We will also test if the most isolated individuals (those who have fewer and farther connections to both people and places) will be most likely to engage in both illicit drug use overall and chemsex specifically, as we believe substance use might serve as a coping mechanism. Further, we will examine differences in exposure to risk, specifically if substance using individuals are more likely to be connected to other substance using individuals as well as more likely to be connected to places with high rates of patron drug use.

To examine H3 and generalize about different demographics’ social networks and place networks, we will fit a series of bipartite exponential random graph models (ERGM) with ego attributes. An ERGM is a statistical model that accounts for a network’s overall structure by describing the propensity of individual nodes to connect based on their individual properties, their shared properties, and their positions relative to one another (60). Bipartite ERGMs include two different node types–in our case our study participant connections to places. We will produce bipartite ERGMs per city for connections to physical venues, and one non-city-specific ERGM for participant connections to online venues. Using these ERGMs, we will be able to assess statistically the observed patterns of collocation across venues, examining differences in clustering and segregation across demographics. Specifically, segregation will be examined through testing of two-star attribute effects ([*Attribute*]*-Match-SP2*) (61) that will investigate if people of the same race/ethnicity, gender, and SES tend to co-attend the same venue. Further, clustering will be examined through testing four-cycle attribute effects (*[Attribute]-C4A2*) (61) that will investigate if racial and gender minorities co-attend the same venues to a greater extent than majority populations. While these models will provide an important initial empirical analysis which may demonstrate structural inequities by identity, in isolation they will be limited in their ability to link structural inequities as a driver of disparities in HIV.

#### 2.4.2 Aim 2: examining structural inequity as a fundamental driver of disparities in HIV and substance use

In aim 2, we will utilize simulation and network modeling across each city to test if structural inequities across social contextual structures account for disparities in HIV, over and above identity alone. We hypothesize that: *(**H1**) venue co-location and (**H2**) fewer social connections to other YMSM-TW will amplify HIV disparities in marginalized populations. Also, (**H3**) those with fewer social connections to other YMSM-TW will be most likely to use substances during sex*.

##### 2.4.2.1 Synthetic populations

Based on data collected using our survey instrument, we will develop a synthetic population for each of the five cities studied to serve as the starting points of our network-based simulations of HIV epidemics. Each city’s synthetic population will match the best empirical estimates available for the number of YMSM-TW aged 18–29 residing in that city. Further, we will ensure that these synthetic populations correctly match the demographic makeup of their empirical counterparts with respect to four racial/ethnic groups (Black, Hispanic/Latinx, non-Hispanic/Latinx White, and Other), two age groups divided at legal drinking age (under 21, 21–29), and two categories of gender identification (cisgender, transgender). Within each city’s synthetic population, we will match simulated YMSM-TW individuals–“synthetic egos”–to empirical YMSM-TW in our data who match according to city of residence, age group, race/ethnicity, and gender identification. This matching will drive the set of physical and online places the synthetic egos attend in our simulation.

##### 2.4.2.2 Modeling co-presence and sexual relationships

Over the course of our simulations, each member of a synthetic population will encounter other members and potentially form sexual partnerships, based in part on co-presence at physical and online places. Each synthetic ego in the population is assigned an empirical counterpart in our original empirical data according to shared city of residence, age group membership, race/ethnicity, and gender identification. With this pairing complete, we will stochastically assign synthetic egos to places over timesteps of a week based on the historical attendance frequencies of their empirical counterparts. This results in a record of co-located synthetic egos each week, per simulation run. We will pass this simulated attendance output to the network-based component of our model, described below. In turn, the network-based component of our model increases the likelihood of sexual partnership formation between two synthetic egos from their base likelihood when they have recently co-located in one or more interaction venues.

##### 2.4.2.3 TERGM fitting

Our simulations will use temporal exponential random graph models (TERGMs) to represent the broader network of sexual partnerships within a given city and the formation and dissolution of individual partnerships over time. These TERGMs include a dynamic network component that accounts for the typical duration of specific types of sexual partnerships between YMSM-TW. Following the sexual partnership stratification conventions of past HIV modeling work (62–66), we will stratify sexual partnerships into three types: longer-term “serious” partnerships, shorter-term “casual” partnerships, and one-time partnerships lasting a single time step. We will fit a TERGM for each of these partnership types, allowing synthetic egos to form and dissolve different kinds of partnerships in parallel.

At each time step, the TERGMs in our simulations determine the likelihood that two previously unpartnered synthetic egos will form a new partnership. This likelihood is determined by the set of cross-sectional target statistics derived from our empirical data, which accounts for factors such as each potential partner’s age, race/ethnicity, gender identification, recent co-location at venues, and number of ongoing (thus concurrent) sexual partnerships. For ongoing serious and casual partnerships, corresponding TERGMs also determine whether a partnership will dissolve stochastically with an average duration for each type of partnership derived from the empirical data.

While TERGMs have been used to simulate sexual partnerships in the past, our inclusion of venue co-location as a formative factor is particularly novel. Our TERGMs incorporate co-location using a fuzzy node-match model term, specially-developed in collaboration with the EpiModel team (67), that is itself an innovation in the fitting of TERGMs. The traditional node-match term uses exact attribute matches. The fuzzy node-match allows for node-matching to occur where there are multiple data points in a single attribute. The fuzzy node-match term will be a “match” based on the individual venues in each of the sets.

##### 2.4.2.4 Stochastic network epidemic simulation

Our simulations of HIV epidemics will account for various forms of social complexity by simulating sexual partnership dynamics and individual movements between venues. Our ability to handle this complexity depends on our use of Repast4Py for parallel processing of agent co-location (68, 69), and on the *EpiModel* software framework (67) for constructing stochastic network epidemic models. Widely used and well-validated, the *EpiModel* framework allows scholars to continuously simulate TERGM-based sexual partnership networks while also accounting for sexual behavior within individual partnerships, intrahost viral dynamics, individuals’ access to treatment across the prevention-care continuum, and interhost epidemiology (i.e., viral transmission). This will allow our simulations to model and account for the effects of resources and behaviors known to shape HIV transmission dynamics such as condom use.

*EpiModel* is thoroughly customizable, allowing scholars to incorporate new phenomena and explore their effects on epidemics. Our simulations will capitalize on this flexibility by modifying EpiModel to interface with our team’s chiSIM framework, thereby making co-location at venues a contributing factor in partnership formation. Additionally, we will modify *EpiModel* to incorporate sexualized substance use (“chemsex”) as a contributing factor to HIV epidemics. Using the best empirical estimates available, informed by the data on substance use we will collect via Network Canvas, we will model the likelihood that pairs of sexual partners engage in sexualized substance use and whether this results in modified rates of condom use. We will also modify *EpiModel* to account for potential reductions in the efficacy of HIV treatment resulting from substance use.

We will calibrate the per-act transmission probability (a global parameter) in our EpiModel models to local prevalence data and generate uncertainty bounds. We take a Bayesian calibration approach, using Approximate Bayesian Computation (ABC) implemented with sequential Monte Carlo sampling (70), facilitated by the high-performance computing model exploration framework EMEWS (71, 72). These calibration methods are robust methods to ensure alignment between network epidemic models and local epidemics (73).

We will analyze the sensitivity of our key output variables, absolute and relative disparity by race/ethnicity and gender minority status in HIV incidence, to the effects of social structure (e.g., physical venue attendance or online app use) via one-at-a-time sampling (Morris Method) (74). We will also investigate global parameter interactions via polynomial chaos expansion (75) and adaptive, surrogate-based methods (76). For all our key output variable estimates, we will produce uncertainty estimates based on estimated measurement and sampling error in the empirically-estimated sexual network and behavioral parameters that serve as simulation inputs.

Using the resulting calibrated models, we will test the impact of structural inequities in (H1) venue co-location and (H2) group differences in social/sexual network size, as well as (H3) differences in rates of substance use during sexual encounters, among other variables, on simulated HIV disparities. In particular, we anticipate that racial and gender disparities in HIV will be increased by these three factors.

#### 2.4.3 Aim 3: conduct city-specific analyses

We will explore differences in the experiences of populations across cities. Given the literature which suggests that movement to urban centers is a way for marginalized populations to cope and find community (77, 78) we will capture data on ego being born in or later moving to (born in the specified city, *native*; moved into the city as an adult, *non-native*) and incorporate Ego nativity as a predictor. We hypothesize that: *(**H1**) metrics of structural inequity might be amplified by the proportion of non-native residents.*

Further, we will utilize our models to identify potential local targets for public health intervention. Public health organizations may seek to determine the optimal location for place-based interventions to minimize new HIV infections in their jurisdiction, given constrained funds, resources, and timelines. These possible interventions include condom distribution (79, 80), pop-up HIV testing clinics (81, 82), and outreach about PrEP (83, 84), whether through in-person engagement, printed informational materials, or geo-fenced online advertisements (85). For each city, we will run optimization experiments to minimize HIV infections over different selections of local physical and online venues (from the list of venues named by participants in the network interviews) selected for intervention given a set intervention budget, assuming constant intervention response rates. This work of determining feasible place-based intervention sets will be guided by close collaboration with local public health and our local community advisory board members. More generally, analysis results about the local structural factors contributing to elevated HIV rates among marginalized populations may inform how local public health officials integrate social determinants of health considerations in their broader health strategies, as well as their outreach tactics and engagement with local community organizations (86–88).

## 3 Discussion

Our SILOS protocol bridges multiple scales, from the individual to their personal network, and from personal networks to city-wide networks. It zooms out spatially, from individual’s social and health care places, to city-wide, to a cross-city comparison. It also converts snapshots-in-time from interviews to a dynamic model of partnership formation and disease dynamics over time. The scale-crossing of this protocol makes it especially promising to investigate structural health disparities visible in aggregate but potentially driven through the interplay of individuals’ varying levels of social access, support, and stigma alongside behavior.

This scale-crossing is powered by the large-sample, rich empirical data collection enabled through self-administered interviews–a new capability–on Network Canvas. Network Canvas, through NIH funding, was specifically designed to simplify the collection and storage of complex network data. Through its visual approach, tactility, micro-interactions, and physical metaphor, Network Canvas enables study participants to efficiently build and interact with their own network.

While this project is certainly not the first to study networks in the context of MSM and HIV, to our knowledge, our inclusion of sexual networks, social support networks, and place networks in one study is novel to this area. This will likely advance our understanding of the relative importance of these networks, and the multiplicative or moderating effects of social isolation, on HIV disparities for multiple-marginalized YMSM-TW.

This project is also conceptually novel in its consideration of how stigma processes may act as an underlying fundamental factor which concentrates disease within certain subpopulations. Too few researchers have considered stigma itself as a fundamental cause of health disparities that impacts both racial and sexual and gender minority populations in similar ways (89).

Interpreting how stigma manifests in each city’s particular YMSM-TW racial/ethnic communities will require not only robust Network Canvas data, but robust research engagement of our CABs. Indeed, a key strength of this proposal is the central research role of CABs: their intended contributions extend far beyond outreach.

To date, relatively little work has incorporated participatory modeling into simulations of infectious disease transmission with representative datasets of marginalized populations. Usually, at best, research protocols in this space focus on two out of three quantitative disparities research needs: engaging participation of the subpopulation studied (90, 91), scaling to general public health dynamics, or appealing to the statistical power of the subpopulation’s representation. Participatory agent-based modeling approaches may engage the study population via modeling workshops [e.g., (92)], and result in a simulation that can scale to broader dynamics theory, but may struggle with statistical representativeness of that subpopulation; network models can incorporate empirical administrative data on subpopulations of interest and scale to the general population, but might not meaningfully incorporate the lived experience of the subpopulation studied. Detailed qualitative interviews and focus panels validated with administrative data [e.g., (93)] likely have an advantage in representing individuals’ experience content-wise and statistically, but do not facilitate analysis of broader public health dynamics. Our protocol attempts to re-balance these three competing needs, with a clear relationship between the computational modeling, the quantitative data collection, and meaningful community collaboration, using multiple sources of knowledge to evaluate hypotheses about the drivers of HIV disparities.

Our focus on meaningful community collaboration is both innovative and necessary. This study builds on our team’s established history of collaboration with local community organizations–local public health departments (94), community-based organizations, and community advisory boards whose members are recruited with the intention of promoting diverse perspectives.

Already, the CAB from our previous Chicago-centric research project have contributed to this protocol. The CAB members directed the selection of the five cities in this study. They hypothesized, based on their experience, of certain cities attracting migration of sexual and gender minorities, and the effect of that influx on the experience of racial/ethnic minoritized populations in those cities. The cities were specifically selected to facilitate a comparison on this axis in our future analyses.

As in our previous work, we anticipate the CABs will inform the network interview instrument design and help interpret responses with local context and identity-based or socioeconomic status-specific experience. For example, our initial network interviews focused on physical, formal venues like bars or clubs for meeting sexual partners; CAB members contributed the importance of key social events (which may even rotate location) for subpopulations, as well as the importance of certain street corners, parks, or subway lines, and other public spaces that have lower barriers of entry.

Our study and protocol face several challenges and limitations in evaluating our hypotheses.

Most critically, studies like this require sizable interdisciplinary teams with sustained effort over several years, traditionally funded by competitive NIH R01 grants. Our study team was awarded a five-year, $3.8 million grant in August 2024. We were notified on 14 March 2025 that our grant had been abruptly terminated by the NIH, among hundreds of other projects in health disparities research, claiming that the “award no longer effectuates agency priorities.” We appealed the termination but received no indication that the appeal was being evaluated. Currently, industry and non-profit foundations do not offer grants of this magnitude for singular observational research studies on social drivers of health disparities, posing a major limitation to adopting this type of study design. Optimistically, on 1 August 2025, the US District Court delivered a preliminary injunction to our PI, Michelle Birkett, among other formerly NIH-funded PIs in LGBTQ health research, blocking the termination and ordering its restoration. Still, we have yet to hear from the NIH on any restoration of the award.

In the meantime, we are considering contingency plans for downscaling the research project. One, we may reduce the number of cities considered. We may also limit the cost of network interview compensation by reducing the network interview recruitment targets, reducing the time burden of the network interviews by re-scoping the instrument, and in turn reducing the compensation incentive. This was suggested by our CAB, who offered that, given the grant terminations targeting LGBTQ health research, potential recruits may be motivated to contribute to the research project as volunteer labor. In this way, the CAB is already contributing to contingency plans. We are waiting on greater clarity on our grant’s status before recruiting participants for the network interviews, whether at the initial scale or downscaled.

Elsewhere, this protocol carries common limitations of survey instruments relying on human recall and self-reporting–sample bias, recall bias, social desirability bias and the effect of stigma. While the sample population is sensitive to differential participation across demographics, the rolling recruitment design allows for dynamic adjustment of targeted recruitment tactics. In addition to the assessing the representativeness of the demographics of respondents, we can assess the geographic coverage of reported places attended against the CABs’ local knowledge or external data on neighborhoods catering to MSM. The CABs, as their members are comprised of the study population, will provide strategies on recruitment to compensate for differential enrollment.

The effect of stigma on the data collection is more challenging to address via dynamic recruitment. It is especially of concern if, as we hypothesize, stigma is correlated with marginalized identity, confounding comparisons across identities. Issues related to stigma can potentially arise in recruitment and sampling if MSM with stronger feelings of stigma are more reluctant to participate in our study. Omission of these participants would bias the set of physical and online venues collected by our instrument; further, networks used to inform our epidemic models would reflect sexual and substance use partnerships of less vulnerable MSM, limiting the ability of our models to inform interventions for those with the greatest need. While we acknowledge the potential for stigma-related biases, our study design mitigates these issues by employing self-administered network interviews and by refraining from recording participant names (and the names of their social ties) during data collection. We anticipate that these design choices will reduce reluctance to participate and elicit more complete and representative responses. Indeed, past work has shown that remote network interviews may suffer less from social desirability biases than those conducted face-to-face (95, 96). Even when sampling, stigma, and social desirability biases are assuaged, network interviews may still suffer from recall biases during data collection, which we mitigate in two ways. We adhere to “content-based name generator questions” (97) based on concrete shared behaviors instead of subjective feelings whose interpretations may vary over time (98). We also restrict the questions to behaviors in the 6 months prior to the interview.

Despite the depth and breadth of the proposed data collection, there are limits in the claims its analysis can make about causality among place-attendance patterns, sexual behaviors, and social networks. The proposed data collection is a point-in-time snapshot, not a longitudinal survey, and it is observational only. Therefore, we can only demonstrate static associations between respondents’ place-attendance patterns, sexual behaviors, and social networks; the network data collection will not inform how changes in a sexual behavior influences one’s place-attendance pattern, or vice versa. Still, information on co-occurrence of behaviors and place attendance still serves the purpose of tailoring public health outreach in conditions similar to those observed in the study.

Where our study can make causal claims is in the relationship between sexual partnership formation patterns and epidemiological outcomes. Since EpiModelHIV simulates a causal process with the partnership networks as input, and calculates resulting epidemiological outcomes, we can run simulation experiments causally linking partnership formation patterns to epidemiological patterns. We consider different place co-attendance patterns as “treatments,” and through our network edge calibration process (which we developed in previous work), hold all other epidemiologically input parameters constant (e.g., total number of partnerships, population size per demographic group, condom use, ART uptake), such that the epidemiological effect of the co-attendance pattern is isolated. Assuming the validity of EpiModelHIV, these simulation results generate causal evidence of the epidemiological effects of the sexual partnership patterns explored.

To evaluate the more ambitious claim that these effects explain empirically observed HIV rates, we would want to additionally evaluate this model’s output’s fit to the observed HIV rates compared to other leading explanations, taking into account the relative simplicity/complexity of the explanations, or evaluate their output on unseen data (99).

While this computational experiment approach does model the epidemiological effect of network properties, it does not model feedback from the epidemiolocal output to re-wire the partnership network. In other words, we do not explicitly model how new HIV diagnoses, or the perception of HIV hotspots, might change agents’ place attendance patterns. For our simulations, we assume that this feedback has negligible effect on the timescales studied.

Lastly, the computational techniques proposed, while fully specifiable and generally repeatable, still require human modeler involvement in the calibration process. Fitting ERGMs to match co-location rates and other data informing relationship formation requires some manual fine-tuning of parameter weights until the fitting algorithm converges on a stable network model. However, the general sensitivity of ERGMs mitigates concerns of overfitting: excessive model specification does not necessarily produce a stable, converging network model, let alone one that successfully matches the target statistics.

In general, deciding which features to incorporate into an ABM is an unbounded problem; some decisions on the time scales, the set of behaviors, and other considerations are more justifiable than others, but reasonable modelers may differ in their choices of ABM specification. This project errs on the side of “keep it as simple as possible, but no simpler”–in other words, the minimal set of attributes to evaluate our hypotheses (99) and still reproduce in simulation the HIV disparities we are studying. In practice, this means keeping the possible agent actions limited to attendance of specific places and the behaviors already modeled in EpiModelHIV (such as condom use and antiretroviral therapy uptake), unless we encounter significant HIV trends among our subject populations that we are unable to explain with our model assumptions. The co-presence model does not introduce any free behavioral parameters to calibrate or intuit; instead, the attendances are directly sampled from the empirical egos. Between the empirically-sampled co-presence model and well-precedented network HIV model, our composite model allows us to proceed conservatively from empirical data and previous work without inviting many more parameters prone to overfitting or reliant on human judgment. Still, if we ultimately entertain modeling additional relevant behaviors based on, for example, CAB input, which would be sensitive to the representativeness of the CAB, participatory protocols like this one could always benefit from the introduction of objective criteria in future model feature selection and validation, borrowing from developments in, for example, the field of inverse generative social science (100).

Overall, this paper describes a protocol for an innovative observational research study across five US cities that will better our understanding of the social contexts of racial, ethnic, and sexual and gender minorities, as well as how inequities in social contexts may drive HIV and substance abuse. Whether or not we will be able to execute SILOS in its original form given the current funding limitations is unclear, but protocols like this that integrate rich network interviews, deep community engagement, and rigorous computational modeling appropriately match the complexity of health disparities that may operate on individual, neighborhood, and structural scales. We hope this project, if nothing else, is able to serve as a model for the kind of systems-informed approach necessary to transform scientific understanding of the structural drivers of health inequity.

## 4 Ethics and dissemination

To facilitate dissemination to a wide audience, we plan to produce a strong web presence. We will also utilize our models to identify tangible local targets for public health intervention. We will share these analyses with local government public health departments and their trusted community partner organizations only.

Due to our commitment that our work has tangible impact, we will create a web presence which will serve as an online hub in which to disseminate our modeling work to both technical and non-technical audiences.

For technical audiences, the model and workflows will be made available through publicly accessible repositories. Furthermore, all (fully deidentified) model parameters will also be shared on public repositories.

For non-technical audiences, our website will include city-specific visualizations that will help communicate our findings in intuitive and tangible ways to public health officials, to policy makers, and to the broader research community.

Given stigmatization concerns–including the stigmatization of individuals who attend the identified sites, as well as potential broader stigmatization if the sites are perceived as associated with a particular community, demographic, or subculture–only examples of the broader city’s HIV dynamics will be displayed on the online hub, not the identification of specific sites or Census blocks.

Recognizing the sensitive subject matter and the potential vulnerability of the marginalized populations participating in the network interviews, we have obtained a Certificate of Confidentiality from NIH to assure confidentiality of study participants’ data. Interview data will be stored in a password-protected, encrypted database accessible only to the research team and, if necessary, the IRB. Any identifiable information from the consenting process will be stored, password-protected, separately from the research data.

The SILOS data collection has been approved by the Northwestern University Social & Behavioral Sciences IRB (STU00221768).

## Supplementary material

Supplementary Data Sheet 1 Summary of draft Network Canvas survey instrument.

Supplementary file 1 Draft Network Canvas survey instrument (Network Canvas file).
