# Supplementary material for: Understanding social context in HIV and drug use via community-informed computational modeling: SILOS study protocol
Source: Front Public Health. 2025 Dec 4;13:1690612. doi: 10.3389/fpubh.2025.1690612 (PMC12711470; doi:10.3389/fpubh.2025.1690612)
Supplement: Supplementary Data Sheet 1 — Summary of draft Network Canvas survey instrument. [file Data_Sheet_1.pdf]

sample network interview instrument.netcanvas

Draft of the network interview instrument described in "Understanding Social Context in HIV and Drug Use via Community-Informed Computational Modeling: SILOS Study Protocol"

LAST MODIFIED:3/7/2025, 10:32:19 AM

SCHEMA VERSION:7

DOCUMENT CREATED: 10/30/2025 12:18:03 PM

Contents

STAGES

1. Welcome
2. Ego Information
3. Name Generator Instructions
4. All Name Generators
5. Sociogram, drug, and sex partners
6. Ordinal Bins
7. Categorical Bins
8. Ego Substances
9. Sex categorical bin
10. Condom categorical bin
11. Vaginal Sex
12. Vaginal Categorical Bin
13. Alter Substances
14. Sex Partner Form
15. Sex Partner Place Met
16. Poppers
17. Methamphetamine
18. Sex sociogram
19. Completion Code

CODEBOOK

Ego

NODE TYPES

Person

EDGE TYPES

Close

SexTie

ASSETS

VIDEO

node_creation.mov

node_gen2.mov

node_gen3.mov

namegen_updated.mov

1 Welcome

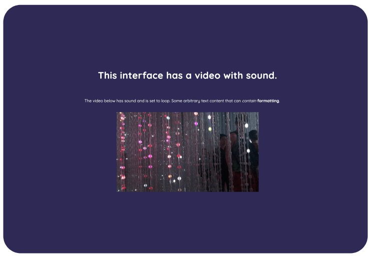

PAGE HEADING

Welcome!

ITEMS

**BLOCK SIZE**	SMALL
**TYPE**	Text
**CONTENT**	To complete the BARF survey, please answer the questions presented on each stage until you reach the end. *The estimated time for completing this survey is between 20-30 minutes*.<br><br>At the end of the survey, after you reach the completion code, a final stage will appear where you will click the green 'Finish' button to record your answers as complete.<br><br>**Note: this survey will not timeout, but if you close the window and relaunch the survey, your data (i.e., answers) will not be saved and you will have to start again.**

INTERVIEWER SCRIPT

2 Ego Information

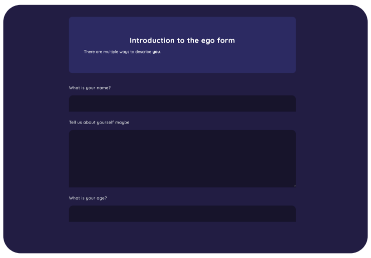

INTRODUCTION PANEL

About You

To get started, please tell us a bit about yourself

FORM

VARIABLE	COMPONENT	PROMPT
EgoGender	RadioGroup	How would you currently identify your gender?
EgoHispanic	Boolean	Please specify if you identify as Hispanic/Latino
EgoRace	CheckboxGroup	Please specify how you describe your race or ethnic background (check all that apply)
YearsLived	Number	How many years have you lived in Chicago?

INTERVIEWER SCRIPT

3 Name Generator Instructions

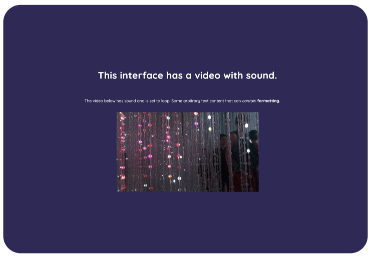

PAGE HEADING

People in Your Life

ITEMS

BLOCK SIZE	MEDIUM
TYPE	Text
CONTENT	On the next screen, we will ask you about the people you have interacted with in the last six months. To add an individual in response to a prompt, press the **‘Add a Person’ Icon** at the bottom right corner of the screen. Type initials, a nickname, or a unique phrase that will help you remember this person along with their age and press Enter.<br><br>After the first prompt, you can choose people you added from previous prompts by clicking on them in the left side panel and dragging them into the main panel.<br><br>The video below offers a preview of how the next screen will work. Please continue to the next screen once you feel comfortable with how to add people.

NAME	namegen_updated.mov
BLOCK SIZE	MEDIUM
TYPE	Video
DURATION	56.85s
PREVIEW	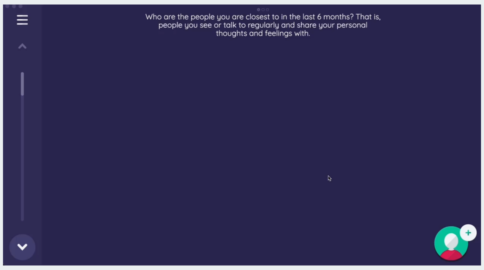

4 All Name Generators

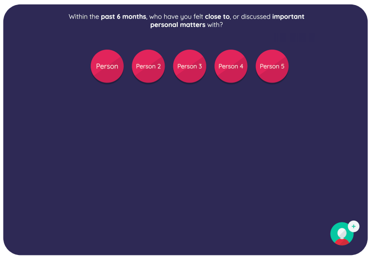

SUBJECT	Person
VARIABLES	addedClose, addedDrugs, addedSex, Age, Close, Drugs, name, Sex

PANELS

1.

TITLE	People you have already listed
DATA SOURCE	*Existing network*

PROMPTS

1. Who are the people you have felt close to in the past 6 months? That is, people you see or talk to regularly and share your personal thoughts and feelings with.

VARIABLE	VALUE
Close	*TRUE*
addedClose	*TRUE*

2. Who are the people that you have used marijuana or other drugs with in the past 6 months? You can drag and drop people you've already mentioned from the side panel or add new people.

VARIABLE	VALUE
Drugs	*TRUE*
addedDrugs	*TRUE*

3. Who are the people that you have had sex with in the past 6 months? You can drag and drop people you've already mentioned from the side panel or add new people.

VARIABLE	VALUE
Sex	*TRUE*
addedSex	*TRUE*

FORM

Title: Name

VARIABLE	COMPONENT	PROMPT
name	Text	**Without using their actual name**, who is this person? Please provide initials, a nickname, or a unique phrase that will help you remember this person. Please do not use the same label for more than one person.
Age	Number	What is this person's age?

BEHAVIOURS

MINIMUM NODES ON STAGE	1

INTERVIEWER SCRIPT

5 Sociogram, drug, and sex partners

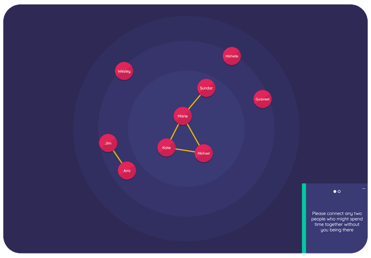

SUBJECT	Person
VARIABLES	Cords, Drugs, Serious, Sex

PROMPTS

1. Connect any two people you know who are very close or spent time together in the past 6 months. You can connect two people by clicking on them one after the other.

LAYOUT VARIABLE	Cords
CREATES EDGE	Close
ALLOW HIGHLIGHTING	*FALSE*

2. Tap on all of the people you have used drugs with in the past 6 months. Anyone you mentioned previously will appear highlighted and does not need to be tapped again.

LAYOUT VARIABLE	Cords
ALLOW HIGHLIGHTING	*TRUE*
HIGHLIGHT VARIABLE	Drugs

3. Tap on all of the people you have had sex with in the past 6 months. Anyone you mentioned previously will appear highlighted and does not need to be tapped again.

LAYOUT VARIABLE	Cords
ALLOW HIGHLIGHTING	*TRUE*
HIGHLIGHT VARIABLE	Sex

4. Please tap on anyone you are currently in a serious relationship with

LAYOUT VARIABLE	Cords
ALLOW HIGHLIGHTING	*TRUE*
HIGHLIGHT VARIABLE	Serious

BEHAVIOURS

AUTOMATIC LAYOUT ENABLED	*TRUE*

INTERVIEWER SCRIPT

6 Ordinal Bins

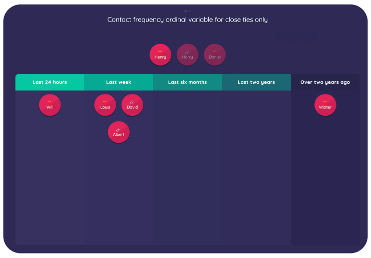

SUBJECT	Person
VARIABLES	RelStrength

PROMPTS

1. Rate the strength of your relationship with each person

VARIABLE	RelStrength

INTERVIEWER SCRIPT

7 Categorical Bins

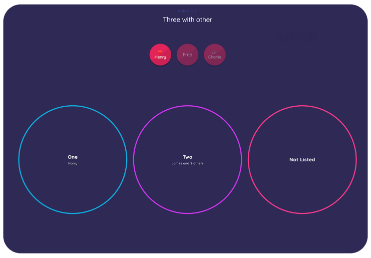

SUBJECT	Person
VARIABLES	Gender, Hispanic, otherRace, Race, SexOrient

PROMPTS

1. Please specify how each of these people currently identify their gender

VARIABLE	Gender

2. Please specify if each of these people identifies as Hispanic or Latino

VARIABLE	Hispanic

3. Please specify how each of these people describes their race or ethnic background

VARIABLE	Race
OTHER VARIABLE	otherRace
OTHER VARIABLE PROMPT	How does this person describe their race or ethnic background?
OTHER OPTION LABEL	Other

4. Please specify how each of these people describe their sexual identity

VARIABLE	SexOrient

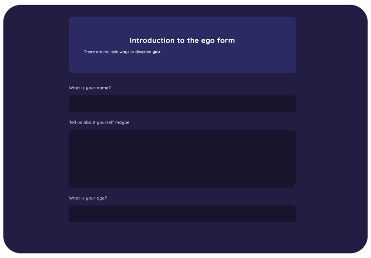

8 Ego Substances

INTRODUCTION PANEL

Please indicate which of the following drugs you have used in the past 6 months

FORM

VARIABLE	COMPONENT	PROMPT
MarijuanaUsed	Boolean	Did you use marijuana in the past six months?
CocaineUsed	Boolean	Did you use cocaine in the past six months?
HeroinUsed	Boolean	Did you use heroin in the past six months?
PainkillersUsed	Boolean	Did you use painkillers or opiates in the past six months?
PoppersUsed	Boolean	Did you use poppers in the past six months?
MethUsed	Boolean	Did you use methamphetamine in the past six months?

9 Sex categorical bin

SUBJECT	Person
VARIABLES	AnalSex, Sex

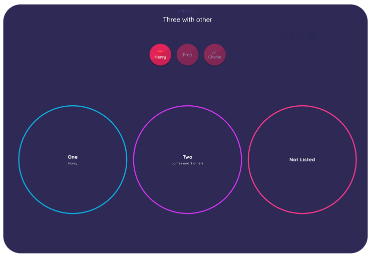

NETWORK FILTERING

RULES Person where Sex is exactly equal to **true**

SKIP LOGIC

ACTION	SHOW
RULES	Person where Sex is exactly equal to **true**

PROMPTS

1. Please indicate if you had anal sex with each sexual partner

VARIABLE	AnalSex

10 Condom categorical bin

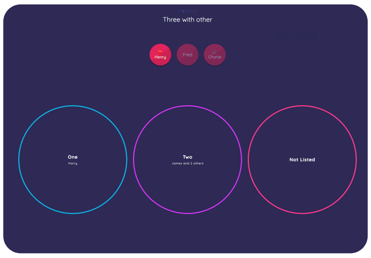

**SUBJECT**	Person
**VARIABLES**	AnalSex, CondomCat, Sex

NETWORK FILTERING

RULES

Person where AnalSex includes **Anal sex**

AND

Person where Sex is exactly equal to **true**

SKIP LOGIC

ACTION SHOW

RULES

Person where AnalSex includes **Anal sex**

AND

Person where Sex is exactly equal to **true**

PROMPTS

1. For each partner, please indicate if you had any anal sex **without** a condom in the past 6 months

VARIABLE CondomCat

11 Vaginal Sex

**SUBJECT**	Person
**VARIABLES**	Gender, Sex, VaginalSex

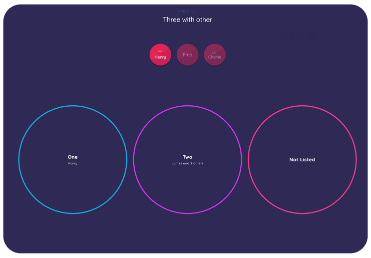

NETWORK FILTERING

RULES

Person where Gender excludes **Cisgender Male**

AND

Person where Sex is exactly equal to **true**

SKIP LOGIC

ACTION SHOW

RULES

Person where Sex is exactly equal to **true**

AND

Person where Gender excludes **Cisgender Male**

PROMPTS

1. Please indicate if you had vaginal sex with each sexual partner

VARIABLE VaginalSex

12 Vaginal Categorical Bin

**SUBJECT**	Person
**VARIABLES**	Sex, VaginalSex, VCondomCat

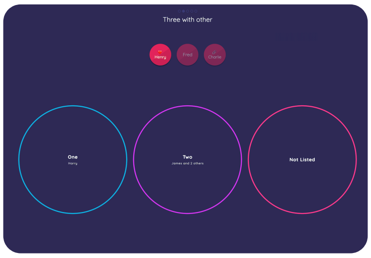

NETWORK FILTERING

RULES

Person where VaginalSex includes **Vaginal sex**

AND

Person where Sex is exactly equal to **true**

SKIP LOGIC

**ACTION**	SHOW

RULES

Person where VaginalSex includes **Vaginal sex**

AND

Person where Sex is exactly equal to **true**

PROMPTS

1. For each partner, please indicate if you had any vaginal sex **without** a condom in the past six months

**VARIABLE**	VCondomCat

13 Alter Substances

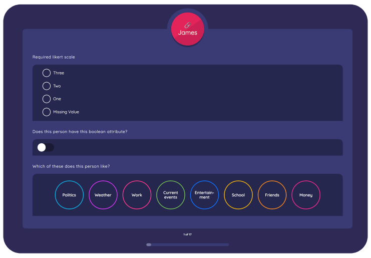

SUBJECT	Person
VARIABLES	AlterDrugsUsed

INTRODUCTION PANEL

Please answer the following questions about each of these people

FORM

VARIABLE	COMPONENT	PROMPT
AlterDrugsUsed	CheckboxGroup	To the best of your knowledge, which of the following substances has this person used in the past 6 months? (Check all that apply)

INTERVIEWER SCRIPT

14 Sex Partner Form

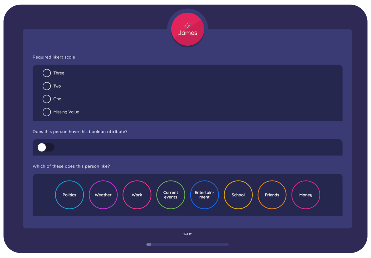

SUBJECT	Person
VARIABLES	FirstSex, LastSex, OngoingPartner, Sex

NETWORK FILTERING

RULES	Person where A/B Sex is exactly equal to **true**

SKIP LOGIC

ACTION	SHOW
RULES	Person where A/B Sex is exactly equal to **true**

INTRODUCTION PANEL

Please answer the following questions about each of your sexual partners

FORM

VARIABLE	COMPONENT	PROMPT
FirstSex	RelativeDatePicker	Please indicate the first day you had sex with this partner. If you don't recall the exact date, provide your best estimate.
LastSex	RelativeDatePicker	Please indicate the last day you had sex with this partner. If you only had sex with this person once, please enter the same date as above in the field below.
OngoingPartner	Toggle	Do you believe you will have sex again with this person in the future?

INTERVIEWER SCRIPT

15 Sex Partner Place Met

**SUBJECT**	Person
**VARIABLES**	OtherPlaceMet, PlaceMet, Sex

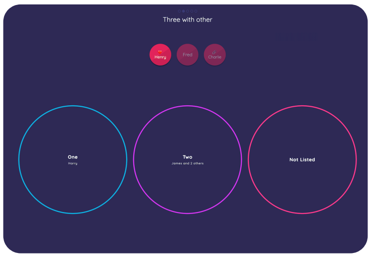

NETWORK FILTERING

**RULES**	Person where Sex is exactly equal to **true**

SKIP LOGIC

**ACTION**	SHOW
**RULES**	Person where Sex is exactly equal to **true**

PROMPTS

1. Where did you meet each of these sexual partners?

**VARIABLE**	PlaceMet
**OTHER VARIABLE**	OtherPlaceMet
**OTHER VARIABLE PROMPT**	Where did you meet this partner?
**OTHER OPTION LABEL**	Somewhere else

16 Poppers

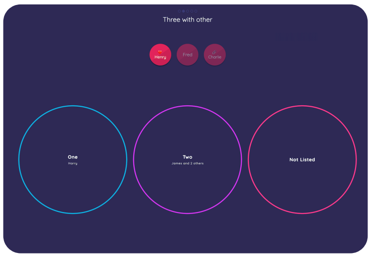

SUBJECT	Person
VARIABLES	chemsexPoppers, PoppersUsed, Sex

NETWORK FILTERING

RULES

Person where Sex is exactly equal to **true**

SKIP LOGIC

ACTION	SHOW

RULES

Ego has PoppersUsed that is exactly equal to **Yes**

AND

Person where Sex is exactly equal to **true**

PROMPTS

1. Earlier you indicated that you had used **poppers** in the past 6 months. Looking back, have you ever used **poppers immediately before or during sex** with each of these partners?

VARIABLE	chemsexPoppers

17 Methamphetamine

SUBJECT	Person
VARIABLES	chemsexMeth, MethUsed, Sex

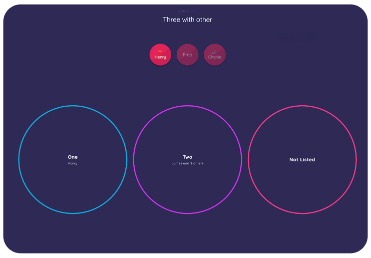

NETWORK FILTERING

RULES	Person where Sex is exactly equal to **true**

SKIP LOGIC

ACTION	SHOW
RULES	Ego has MethUsed that is exactly equal to **Yes**
	AND
	Person where Sex is exactly equal to **true**

PROMPTS

1. Earlier you indicated that you had used **methamphetamine** in the past 6 months. Looking back, have you ever used **methamphetamine immediately before or during sex** with each of these partners?

VARIABLE	chemsexMeth

18 Sex sociogram

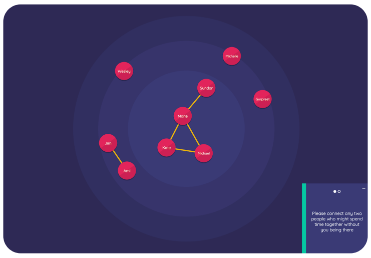

SUBJECT	Person
VARIABLES	Cords, Sex

NETWORK FILTERING

RULES	Person where Sex is exactly equal to **true**

SKIP LOGIC

ACTION	SHOW
RULES	Person where Sex is exactly equal to **true**

PROMPTS

1. Please connect any two sex partners who have had sex with each other in the past 6 months. You can connect two people by clicking on them one after the other.

LAYOUT VARIABLE	Cords
CREATES EDGE	SexTie
ALLOW HIGHLIGHTING	*FALSE*

BEHAVIOURS

AUTOMATIC LAYOUT ENABLED	*TRUE*

19 Completion Code

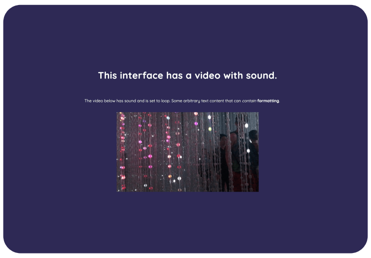

PAGE HEADING

Completion Code

ITEMS

BLOCK SIZE	SMALL
TYPE	Text
CONTENT	Thank you for your participation this the BARF study! To register your completion of this study and receive payment from Prolific, please go to the following URL:<br>https://app.prolific.com/submissions/complete?cc=CUP3XKI0<br>and enter your completion code:

BLOCK SIZE	SMALL
TYPE	Text
CONTENT	**CUP3XKI0**

INTERVIEWER SCRIPT

Ego

Name	Type	Used In
CocaineUsed	boolean<br>VALUE / LABEL<br>*TRUE* / Yes<br>*FALSE* / No	Ego Substances
EgoGender	ordinal<br>VALUE / LABEL<br>Cisgender_Female / Cisgender Female<br>Cisgender_Male / Cisgender Male<br>Transgender_Female / Transgender Female<br>Transgender_Male / Transgender Male<br>Dont_know / Don't know	Ego Information
EgoHispanic	boolean<br>VALUE / LABEL<br>*TRUE* / Yes, Hispanic or Latino<br>*FALSE* / No	Ego Information
EgoRace	categorical<br>VALUE / LABEL<br>Black_African_American / Black/African American<br>American_Indian_or_Alaskan_Native / American Indian or Alaskan Native<br>Asian / Asian<br>White / White<br>Native_Hawaiian_or_Other_Pacific_Islander / Native Hawaiian or Other Pacific Islander	Ego Information

Name	Type	Used In
A/B HeroinUsed	boolean<br>VALUE / LABEL<br>*TRUE* / Yes<br>*FALSE* / No	Ego Substances
A/B MarijuanaUsed	boolean<br>VALUE / LABEL<br>*TRUE* / Yes<br>*FALSE* / No	Ego Substances
A/B MethUsed	boolean<br>VALUE / LABEL<br>*TRUE* / Yes<br>*FALSE* / No	Ego Substances<br>Methamphetamine
A/B PainkillersUsed	boolean<br>VALUE / LABEL<br>*TRUE* / Yes<br>*FALSE* / No	Ego Substances
A/B PoppersUsed	boolean<br>VALUE / LABEL<br>*TRUE* / Yes<br>*FALSE* / No	Ego Substances<br>Poppers
123 YearsLived	number	Ego Information

Person

Name	Type	Used In
addedClose	boolean	All Name Generators
addedDrugs	boolean	All Name Generators
addedSex	boolean	All Name Generators
Age	number	All Name Generators
AlterDrugsUsed	categorical	Alter Substances

VALUE	LABEL
Marijuana	Marijuana
Cocaine	Cocaine
Heroin	Heroin
Painkillers_or_Opiates	Painkillers or Opiates
Poppers	Poppers
Methamphetamine	Methamphetamine
None	None
Dont_know	Don't know/unsure

Name	Type	Used In
AnalSex	categorical	Sex categorical bin<br>Condom categorical bin<br>Condom categorical bin

VALUE	LABEL
Anal_sex	Anal sex
No_anal_sex	No anal sex

Name	Type	Used In
chemsexMeth	categorical	Methamphetamine

VALUE	LABEL
TRUE	Yes
FALSE	No

Name	Type	Used In
chemsexPoppers	categorical<br><br>VALUE / LABEL<br>TRUE / Yes<br>FALSE / No	Poppers
Close	boolean	All Name Generators
CondomCat	categorical<br><br>VALUE / LABEL<br>Condomless_anal_sex / Anal sex without a condom<br>No_condomless_anal_sex / Always used a condom during anal sex	Condom categorical bin
Cords	layout	Sociogram, drug, and sex partners<br>Sociogram, drug, and sex partners<br>Sociogram, drug, and sex partners<br>Sociogram, drug, and sex partners<br>Sex sociogram
Drugs	boolean	All Name Generators<br>Sociogram, drug, and sex partners
FirstSex	datetime	Sex Partner Form

Name	Type	Used In
Gender	categorical<br>VALUE / LABEL:<br>Cisgender_Female — Cisgender Female<br>Cisgender_Male — Cisgender Male<br>Transgender_Female — Transgender Female<br>Transgender_Male — Transgender Male<br>Dont_know — Don't know<br>Not_listed — Not listed	Categorical Bins<br>Vaginal Sex<br>Vaginal Sex
Hispanic	categorical<br>VALUE / LABEL:<br>Hispanic_or_Latino — Yes, Hispanic or Latino<br>Not_Hispanic_or_Latino — No	Categorical Bins
LastSex	datetime	Sex Partner Form
name	text	All Name Generators
OngoingPartner	boolean	Sex Partner Form
OtherPlaceMet	text	Sex Partner Place Met
otherRace	text	Categorical Bins

Gender:

VALUE	LABEL
Cisgender_Female	Cisgender Female
Cisgender_Male	Cisgender Male
Transgender_Female	Transgender Female
Transgender_Male	Transgender Male
Dont_know	Don't know
Not_listed	Not listed

Hispanic:

VALUE	LABEL
Hispanic_or_Latino	Yes, Hispanic or Latino
Not_Hispanic_or_Latino	No

Name	Type	Used In
PlaceMet	categorical	Sex Partner Place Met

VALUE	LABEL
Bar_Club	Bar / Club
Online_Mobile_App	Online / Mobile App
School	School
Work	Work

Name	Type	Used In
Race	categorical	Categorical Bins

VALUE	LABEL
Black_African_American	Black / African American
American_Indian_or_Alaskan_Native	American Indian or Alaskan Native
Asian	Asian
White	White
Native_Hawaiian_or_Other_Pacific_Islander	Native Hawaiian or Other Pacific Islander

Name	Type	Used In
RelStrength	ordinal	Ordinal Bins

VALUE	LABEL
Very_close	Very close
Somewhat_close	Somewhat close
Not_close_at_all	Not close at all

Name	Type	Used In
Serious	boolean	Sociogram, drug, and sex partners

<table>
<tr><th>Name</th><th>Type</th><th>Used In</th></tr>
<tr><td>Sex</td><td>boolean</td><td>All Name Generators
Sociogram, drug, and sex partners
Sex categorical bin
Condom categorical bin
Vaginal Sex
Vaginal Categorical Bin
Sex Partner Form
Sex Partner Place Met
Poppers
Methamphetamine
Sex sociogram
Sex categorical bin
Condom categorical bin
Vaginal Sex
Vaginal Categorical Bin
Sex Partner Form
Sex Partner Place Met
Poppers
Methamphetamine
Sex sociogram</td></tr>
<tr><td>SexOrient</td><td>categorical
<table>
<tr><th>VALUE</th><th>LABEL</th></tr>
<tr><td>Bisexual</td><td>Bisexual</td></tr>
<tr><td>Heterosexual_Straight</td><td>Heterosexual / Straight</td></tr>
<tr><td>Gay_Lesbian</td><td>Gay / Lesbian</td></tr>
<tr><td>Queer</td><td>Queer</td></tr>
<tr><td>Not_listed</td><td>Not listed</td></tr>
<tr><td>Dont_want_to_answer</td><td>Don't want to answer</td></tr>
<tr><td>Dont_know</td><td>Don't know</td></tr>
</table></td><td>Categorical Bins</td></tr>
<tr><td>VaginalSex</td><td>categorical
<table>
<tr><th>VALUE</th><th>LABEL</th></tr>
<tr><td>Vaginal_sex</td><td>Vaginal sex</td></tr>
<tr><td>No_vaginal_sex</td><td>No vaginal sex</td></tr>
</table></td><td>Vaginal Sex
Vaginal Categorical Bin
Vaginal Categorical Bin</td></tr>
</table>

Name	Type	Used In
VCondomCat	categorical	Vaginal Categorical Bin

VALUE	LABEL
Condomless_vaginal_sex	Vaginal sex without a condom
No_condomless_vaginal_sex	Always used a condom during vaginal sex

Close

Name	Type	Used In

No variables to display.

SexTie

Name	Type	Used In

No variables to display.

Resource Library

Video

NAME	node_creation.mov
TYPE	Video
DURATION	34.93s
PREVIEW	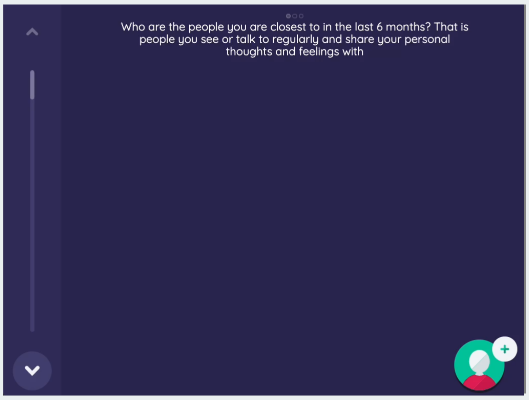

NAME	node_gen2.mov
TYPE	Video
DURATION	58.98s
PREVIEW	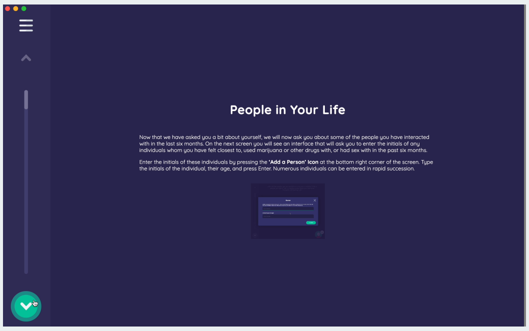

NAME	node_gen3.mov
TYPE	Video
DURATION	58.98s
PREVIEW	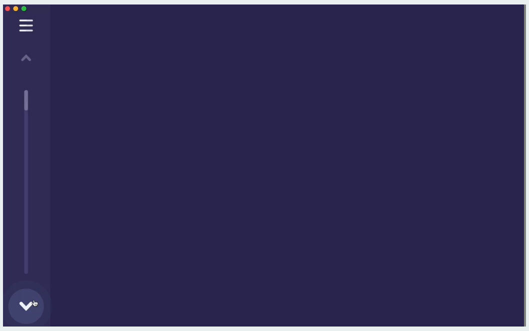

NAME	namegen_updated.mov
TYPE	Video
DURATION	56.85s
PREVIEW	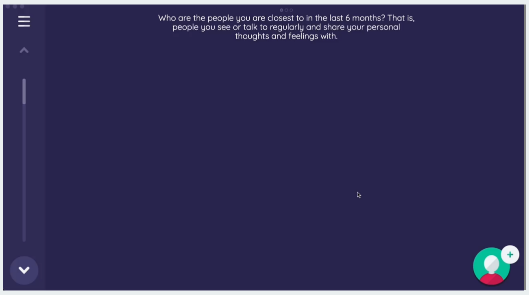
